# TGFβ signaling regulates the choice between pluripotent and neural fates during reprogramming of human urine derived cells

**DOI:** 10.1038/srep22484

**Published:** 2016-03-03

**Authors:** Lihui Wang, Xirui Li, Wenhao Huang, Tiancheng Zhou, Haitao Wang, Aiping Lin, Andrew Paul Hutchins, Zhenghui Su, Qianyu Chen, Duanqing Pei, Guangjin Pan

**Affiliations:** 1 Key Laboratory of Regenerative Biology, South China Institute for Stem Cell Biology and Regenerative Medicine, Guangzhou Institutes of Biomedicine and Health, Chinese Academy of Sciences, Guangzhou, China; 2 Department of Pathology, Medical College, Jinan University, Guangzhou, China; 3 Department of Pathology, Dalian Medical University, Dalian, China

## Abstract

Human urine cells (HUCs) can be reprogrammed into neural progenitor cells (NPCs) or induced pluripotent stem cells (iPSCs) with defined factors and a small molecule cocktail, but the underlying fate choice remains unresolved. Here, through sequential removal of individual compound from small molecule cocktail, we showed that A8301, a TGFβ signaling inhibitor, is sufficient to switch the cell fate from iPSCs into NPCs in OSKM-mediated HUCs reprogramming. However, TGFβ exposure at early stage inhibits HUCs reprogramming by promoting EMT. Base on these data, we developed an optimized approach for generation of NPCs or iPSCs from HUCs with significantly improved efficiency by regulating TGFβ activity at different reprogramming stages. This approach provides a simplified and improved way for HUCs reprogramming, thus would be valuable for banking human iPSCs or NPCs from people with different genetic background.

Neural stem cells (NSCs) or their progenitors (NPCs) and differentiated derivations provide valuable sources in understanding neural diseases and promising strategies to repair CNS (central nervous system) damages^1^,^2^,^3^. Towards these goals, generation of patient’s specific neural cells that are safe and functional is critical. The breakthrough that human somatic cells could be induced back into pluripotent state as iPSCs by reprogramming factors opens up new ways to obtain patient’s specific NPCs for regenerative medicine^4^,^5^. iPSCs could undergo indefinite self-renewal and hold potential to differentiate into most of cell lineages in human body, including neural cells. Indeed, iPSCs from patients with different neural diseases have been reported, for example, Parkinson’s disease^6^,^7^, Huntington’s disease^6^ and Alzheimer’s disease^8^. Further differentiation of the disease specific iPSCs into neural lineage would illuminate our understanding of these diseases mechanistically, as well as provide functional cells for potential autologous transplantation. Alternatively, somatic cells such as fibroblast could be directly converted into NPCs or neurons by retroviral delivery of specific transcription factors or microRNAs^9^,^10^,^11^,^12^,^13^,^14^,^15^,^16^. Without going through the pluripotent state, these so-called induced neural cells might provide good alternative to iPSCs-derived NPCs.

In previous report, we have shown that a small molecule cocktail plus Yamanaka factors could reprogram human urine cells (HUCs) directly into neural progenitor cells (NPCs) rather than iPSCs^17^. These cells could be expanded *in vitro* and differentiate into subtypes of mature neurons and astrocyte cells, providing a convenient approach to generate patient’s specific NPCs suitable for further researches or applications. However, how each small molecule within the cocktail participates in determining the fate choice between iPSCs and NPCs in reprogramming remains unsolved. In current report, we dissected signaling pathways that were responsible for fate decision between iPSCs and NPCs and developed a simplified and improved condition for HUCs reprogramming. Through sequential removal of each individual compound^17^, we showed that A8301, a selective inhibitor for TGFβ signaling was sufficient to switch the cell fate from iPSCs into NPCs in factors (OSKM)-induced HUCs reprogramming. However, our data also showed that early exposure to TGFβ blocks HUCs reprogramming partially by promoting EMT related genes, as reported in other cell types^18^,^19^. Based on these data, we were able to develop an optimized approach for reprogramming of HUCs into NPCs or iPSCs in a defined condition with significantly improved efficiency, which would be valuable for banking human NPCs or iPSCs for future research.

## Results

### A8301 is sufficient to generate UiNPCs from HUCs with OSKM

**Figure 1:**
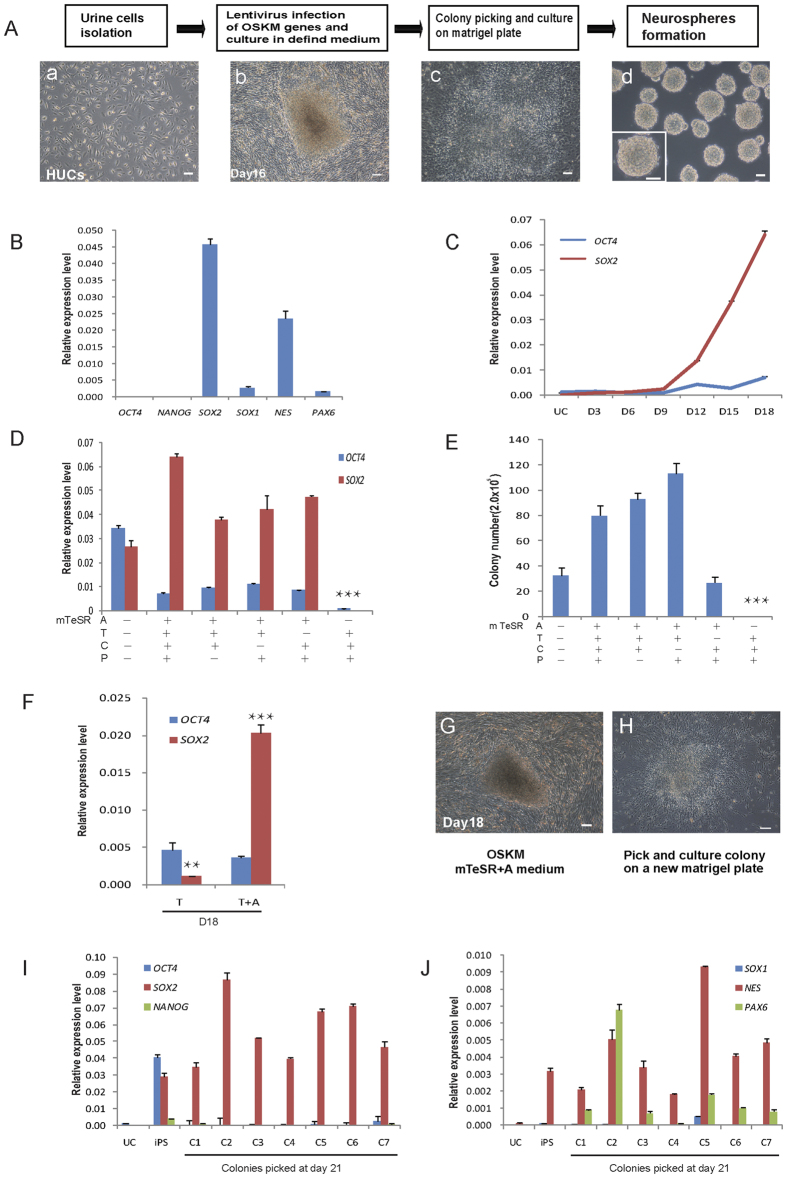
Suppression of TGFβ signaling switches the cell fate during OSKM induced reprogramming of HUCs. (**A**) Generation of NPCs from HUCs. Epithelial-like cells isolated from human urine (a) were transfected with lentivirus vectors encoding reprogramming factors and cultured in defined medium (4i) on Matrigel. Colonies arising at day 16 (b) were picked and re-plated onto Matrigel (**c**) and suspended to form neurospheres (d). (**B**) qRT-PCR analysis of markers expressed in expanded iNPCs (P4). (**C**) Time course qRT-PCR detection of *OCT4* and *SOX2* genes expression. HUCs were reprogrammed in the medium with different signaling pathway inhibitors induced by OSKM, qRT-PCR analysis of pluripotent gene *OCT4* and NSC gene *SOX2* expression (**D**) and the colony number calculation (**E**) suggested that A8301 is indispensable to NPCs generation. HUCs were reprogrammed in mTeSR medium adding A8301 (T + A), and were analyzed *OCT4* and *SOX2* gene expression by qRT-PCR at day 18 (**F**). The dome colonies (**G**) were picked and re-plated onto Matrigel (**H**) or directly analyzed for markers expression by qRT-PCR (**I,J**). The plot shows relative expression levels of the indicated genes in colonies picked at day 21 compared to levels in HUCs and iPSCs (abbreviated as UC and iPS, respectively). Scale bars, 50 μm. Error bars, s.d., based on three replicates (n = 3). *p < 0.05, **p < 0.01, ***p < 0.001.

We have shown that HUCs could be directly converted into NPCs (UiNPCs) by episomal delivery of *OCT4/SOX2/KLF4*/*MicroRNA302-367* plus a cocktail of small molecules (5i, CHIR99021, PD0325901, A8301, thiazovivin, DMH1)^17^. To dissect the role of each small molecule within the cocktail, we re-tested this process using typical viral encoded OSKM (*OCT4/SOX2/KLF4/MYC*) system. We firstly showed that HUCs transduced with lentiviral-OSKM followed with culturing in defined medium containing 4i (ATCP: CHIR99021, PD0325901, A8301, thiazovivin) displayed UiNPCs phenotype. They exhibited rosette like morphology when picked onto monolayer culture and formed typical neurospheres in suspension culture (Fig. 1A). Furthermore, NPC genes such as *SOX2*, *NES* were highly expressed, while pluripotent genes such as *OCT4*, *NANOG* were suppressed (Fig. 1B). Through time course experiment, we showed that *OCT4* was suppressed during the whole process of reprogramming, while *SOX2* was activated (Fig. 1C). These data suggest that the 4i compounds in the medium could also change cell fate of HUCs into NPCs rather than iPSCs. To analyze the role of each compound, we performed OSKM induced HUCs reprogramming in medium with one of each individual compound removed from the 4i cocktail (Fig. 1D and supplementary Fig. S1). By examining the activation of endogenous *OCT4* and *SOX2*, we showed that A8301 was the essential component in the cocktail for UiNPCs generation (Fig. 1D,E). We further demonstrated that adding A8301 alone in reprogramming medium could generate UiNPCs (Fig. 1G,H) which were *OCT4*^−^*/SOX2*^+^ (Fig. 1F). We then randomly picked individual colonies reprogrammed in medium with A8301 alone and confirmed that they expressed very low level of pluripotent genes such as *NANOG* and *OCT4*, but high level of neural genes, such as *SOX2, NES* and *PAX6* (Fig. 1I,J). Together, we demonstrated that A8301, the selective TGFβ signaling inhibitor, is sufficient to switch the cell fate to NPCs during OSKM induced reprogramming of HUCs.

### Generation of UiNPCs with simplified and defined medium

**Figure 2:**
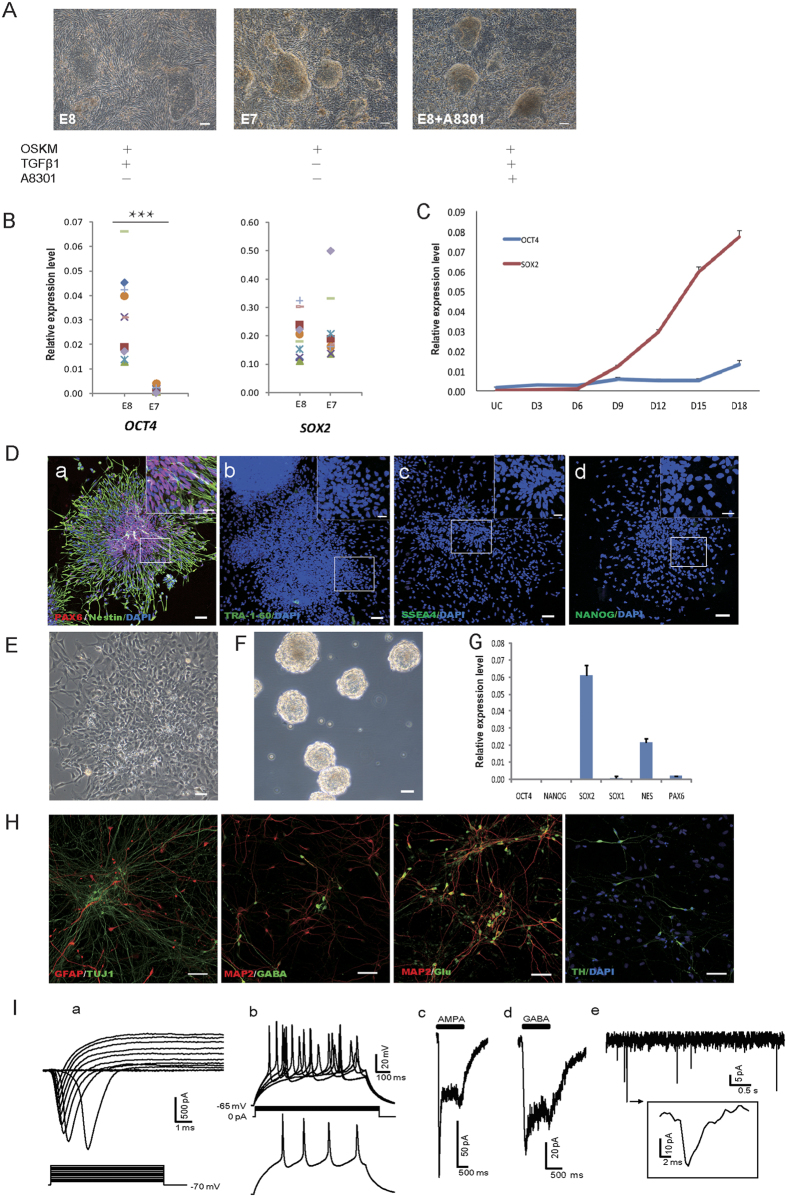
Generation of UiNPCs in simplified medium. HUCs were transfected with lentivirus vectors encoding reprogramming factors and cultured in defined medium E8, E7 or E8 adding A8301 on Matrigel, the morphology of colonies formed in different mediums were different between E8 and E7 or E8 adding A8301 (**A**). The colonies were randomly picked and directly analyzed for *OCT4* and *SOX2* gene expression by qRT-PCR (**B**). The same two genes were detected during the process of reprogramming (**C**). (**D**) Micrographs show immunostains of colonies for the indicated markers (D18). Representative morphology of UiNPCs cultured on Matrigel (**E**) or in suspension as neuospheres (**F**) is shown. (**G**) qRT-PCR analysis of markers expressed by expanded UiNPCs (P5). (**H**) Differentiation of UiNPCs *in vitro*. Immunostains of spontaneously differentiated UiNPCs with antibodies against the indicated markers: GFAP, astrocyte marker; TUJ1, pan-neuronal marker; MAP2, mature neuronal markers; glutamate (Glu), GABA and TH, subtype-specific neuronal markers. (**I**) The electrophysiological properties of UiNPCs derived neurons. (a) Representative voltage-gated ion currents recorded from UiNPC-derived neuron, Whole-cell Na^+^ (inward) and K^+^ (outward) currents (upper panel) were elicited by test pulses to potentials between −50 mV and +60 mV in steps of 10 mV from a holding potential of −70 mV (lower panel). (b) Current-clamp recording showing a representative train of action potentials in a UiNPC-derived neuron (upper panel), intracellular injected currents were step currents incremented by 2 pA from 14 to 22 pA with 800 ms duration (middle panel). Action potentials evoked by 20 pA injected current (lower panel). (c) and (d) showed the current response to AMPA (1 mM) and GABA (1 mM). (e) Representative traces of spontaneous postsynaptic currents (PSCs) recorded from a UiNPC-derived neuron at a holding potential of −70 mV, arrowhead pointed to the magnified view of a typical postsynaptic current. Scale bars: 50 μm; zoom insert in D, 20 μm. Error bars, s.d., n = 3 experiments. *p < 0.05, **p < 0.01, ***p < 0.001.

A concern for using small molecules is that they might have multiple or undefined targets, resulting in unknown genomic or cellular changes. Therefore, we sought to examine whether we could eliminate some small molecules from the cocktail for UiNPCs generation. Chen *et al*. had reported a simplified medium with totally defined condition, called E8 for maintaining human ESCs or iPSCs^20^. E8 medium contains only two growth factors, TGFβ and FGF2 to enable human ESCs self-renewal and E7 medium differs from E8 by removal of TGFβ. We asked whether E7 medium could be used as the reprogramming medium for UiNPCs generation. We transduced OSKM into HUCs and cultured them in medium with or without TGFβ (E8 or E7) (Fig. 2A). As shown in Fig. 2A, dome like UiNPC colonies appeared in both E7 medium (no TGFβ) and E8 medium adding A8301, but not in E8 medium (Fig. 2A). Individual colonies picked from E7 medium expressed very low level of *OCT4*, but high level of *SOX2* (Fig. 2B). Moreover, *OCT4*, not *SOX2* was suppressed during the whole process of HUCs reprogramming in E7 medium (Fig. 2C), indicating that simply removing TGFβ is sufficient for UiNPCs generation. To further characterize UiNPCs generated in E7 medium, we performed immune staining to demonstrate that they were strongly positive for NPC markers like PAX6 and Nestin, but negative for pluripotent markers such as TRA-1–60, SSEA4 and NANOG (Fig. 2D,G). Furthermore, UiNPCs generated in E7 medium could be further expanded in both monolayer and suspension culture condition (Fig. 2E,F) and maintain the potential to differentiate into various subtype neurons and astrocyte (Fig. 2H). More importantly, neurons from UiNPCs generated in E7 medium exhibit typical neuronal connectivity with robust postsynaptic currents (Fig. 2I). Taken together, we demonstrate that HUCs could also give rise to NPCs in simplified defined E7 medium without TGFβ signaling by OKSM reprogramming.

### An improved approach to generate NPCs or iPSCs from HUCs through manipulating TGFβ activity

**Figure 3:**
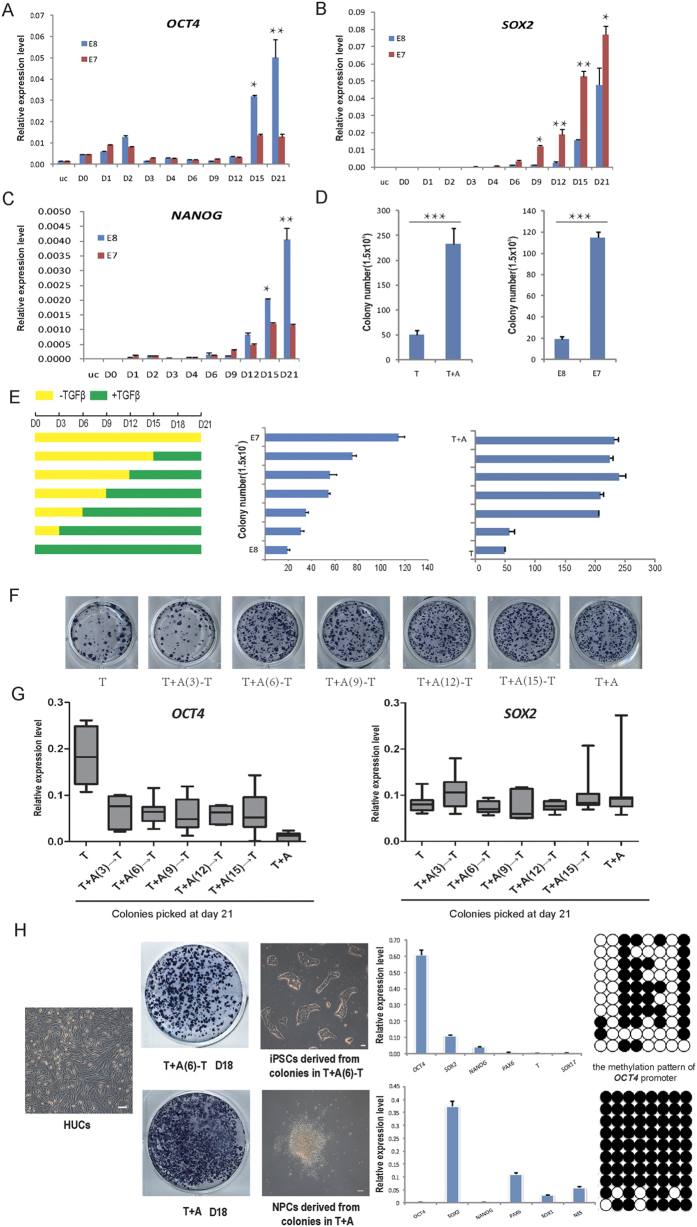
Generation of NPCs or iPSCs from HUCs through manipulating TGFβ activity. qRT-PCR analysis of pluripotent gene *OCT4* (**A**), *NANOG* (**B**) and NSC gene *SOX2* (**C**) expressions in the HUCs cultured in E8 and E7 mediums in the time course of OSKM induced reprogramming. (**D**)The colony-forming number in the two kinds of medium (with TGFβ or no TGFβ) were calculated, the colony number was significantly increased in the medium without TGFβ, which was in sharp contrast to HUCs transfected with the same transcriptional factors but cultured in mTeSR or E8 medium. (**E**) HUCs were performed a systematic reprogramming with introducing TGFβ signaling in different time point including D0, 3, 6, 9, 12, 15, and calculated the colony number formed in different medium at D18 of reprogramming. The colony numbers were calculated. (**F**) The AP staining showed the colony forming in different inducing culture mediums. (**G**) We randomly picked 10 individual colonies from the reprogramming culture medium with different TGFβ exposure time at day 21, detected the *OCT4* and *SOX2* genes expression by qRT-PCR analysis. (**H**) HUCs were reprogrammed by OSKM with inhibition of TGFβ for the first 6 days or the whole process, the colonies were picked and re-plated, and exhibited iPSCs and NPCs morphology with the activation of respective maker genes. DNA methylation profile of the *OCT4* promoters in the indicated iPSCs and NPCs colonies were detected, white circles indicate unmethylated CpGs, and black circles indicate methylated. Scale bars: 50 μm. Error bars, s.d., n = 3 experiments. *p < 0.05, **p < 0.01, ***p < 0.001.

We showed that removal of TGFβ signaling was critical for UiNPCs generation due to the failure of *OCT4* activation (Fig. 2B), suggesting that TGFβ signaling is required for establishing pluripotency during HUCs reprogramming. Indeed, another pluripotent gene *NANOG* also failed to express in the absence of TGFβ. However, *SOX2* activation was not significantly affected by TGFβ, so a dynamic process of reprogramming toward a neural fate occurred in the medium without TGFβ (Fig. 3A–C). On the other side, we noticed that the colony number was much lower in reprogramming medium containing TGFβ (Fig. 3D). These data suggest that TGFβ might suppress HUCs reprogramming during certain time window, even though it’s critical for establishing pluripotency. To optimize the HUCs reprogramming system for high efficiency, we performed a systematic test of both mTeSR and defined E7 medium by introducing TGFβ at the different time point (Fig. 3E). Using E7 medium or mTeSR medium adding TGFβ inhibitor A8301, we showed that early TGFβ exposure significantly suppressed HUCs reprogramming (Fig. 3E,F). The colony number was dramatically increased in the HUCs cultured in the medium with TGFβ addition after D6–9 of reprogramming and reached its peak in the medium without TGFβ (Fig. 3E). We randomly picked colonies reprogrammed with above conditions and analyzed their expression level of endogenous *OCT4* and *SOX2*. As shown in Fig. 3G, TGFβ exposure at later stage of reprogramming is required for the activation of endogenous *OCT4*, but not for *SOX2*. These data indicate that TGFβ plays distinct roles at early and later stage of OSKM induced HUCs reprogramming. While early exposure suppresses reprogramming, later presence is critical for activation of *OCT4* (Fig. 3H) and this determines the cell fate choice of NPCs or iPSCs. This is important because that the generation of human NPCs or iPSCs, particularly using non-vial approaches is usually difficult and inefficient. To test whether this can be applied for routine HUCs reprogramming, we isolated HUCs from other two different donors and performed reprogramming by OSKM with inhibition of TGFβ for the first 6 days or the whole process. We observed efficient generation of iPSCs and NPCs respectively (Fig. 3H). Notably, the iPSCs generation efficiency was significantly improved with this new approach compared with reprogramming in mTeSR medium (Fig. 3F, H and supplementary Fig. S2). The reprogrammed colonies can be successfully picked and maintained as typical human iPSCs or NPCs with the activation of respective maker genes (Fig. 3H). These data demonstrated that we have established an efficient HUCs reprogramming approach through manipulating TGFβ to generate iPSCs and NPCs.

### Exposure of TGFβ activates *SNAI1* that negatively regulate reprogramming and pluripotency

**Figure 4:**
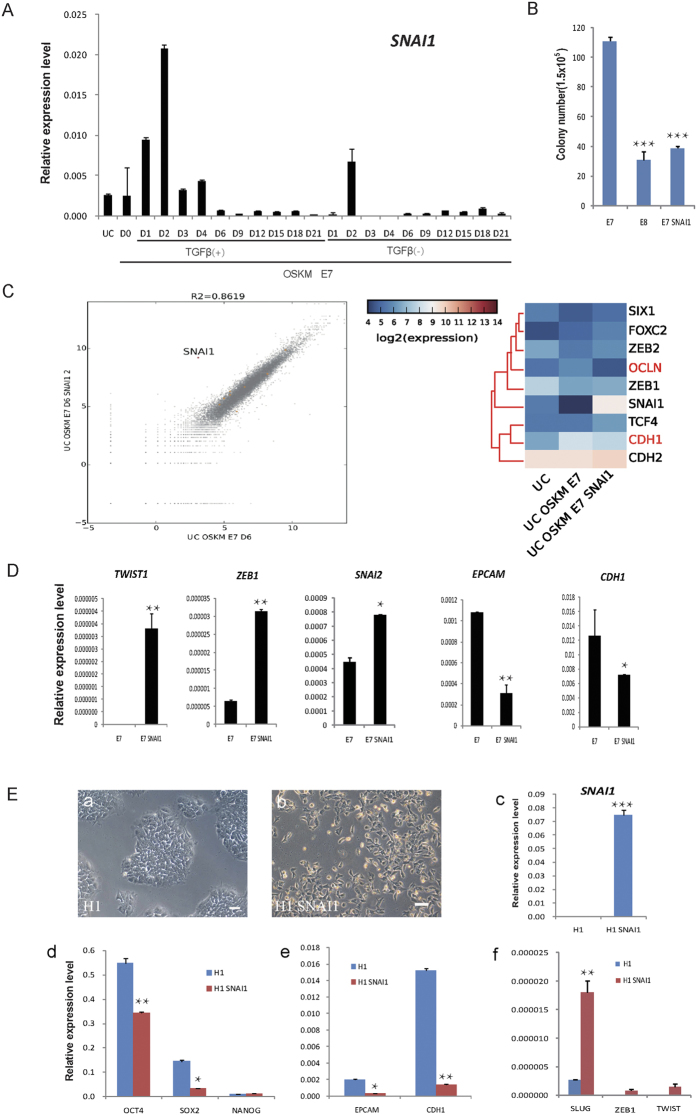
Early exposure of TGFβ activates *SNAI1* that negatively regulate reprogramming and pluripotency. (**A**) The expression of *SNAI1* gene was analyzed in the HUCs cultured in E8 and E7 mediums in the time course of OSKM induced reprogramming. (**B**) The cDNA of *SNAI1* was subcloned into lentivirus vectors and overexpressed by infection in HUCs. The infected cells undergone OSKM factors reprogramming at the same time and cultured in the medium without TGFβ. The colony numbers in different conditions were calculated at D18 of reprogramming. (**C**) Comparison of global gene expression profiles of HUCs cultured in E7 and *SNAI1* overexpressed HUCs cultured in E7 at D6 of reprogramming. R, Pearson correlation coefficient. The heatmap of EMT related genes initiated that mesenchymal related genes (black) were elevated and epithelial genes (red) were decreased in *SNAI1* overexpressed HUCs. (**D**) qRT-PCR results showed the EMT related genes expression in HUCs and the *SNAI1* overexpressed HUCs. (**E**) *SNAI1* was transfected into H1 cells (a, c), cells lost the contraction and the pluripotent morphology (b). The pluripotent gene expressions were significantly down regulated (d). The mesenchymal genes were activated in the *SNAI1* transfected H1 cells (f), however, the epithelial gene expressions were decreased (e). Scale bars: 50 μm. Error bars, s.d., n = 3 experiments. *p < 0.05, **p < 0.01, ***p < 0.001.

It’s known that TGFβ could induce epithelia-mesenchymal transition (EMT), a barrier for somatic reprogramming^18^,^19^,^21^. To elucidate the underlying mechanism that early exposure of TGFβ blocks HUCs reprogramming, we analyzed EMT related genes during the whole process of reprogramming. Among these genes, we found that *SNAI1* was particularly highly activated in the presence of TGFβ (Fig. 4A), suggesting that *SNAI1* might be the key downstream effector of TGFβ at early stage of reprogramming. Indeed, co-transduction of *SNAI1* dramatically suppressed the HUCs reprogramming induced by OSKM (Fig. 4B). Further through RNA-Seq analysis and qRT-PCR, we showed that transfection of *SNAI1* in HUCs dramatically up regulated mesenchymal genes such as *TWIST, SNAI2, ZEB1, and ZEB2*, but suppressed epithelial genes such as *EPCAM* and *CDH1* (Fig. 4C,D). Taken together, these data demonstrate that *SNAI1* promotes EMT of HUCs and further suppress OSKM induced reprogramming. Beside the role of *SNAI1* in reprogramming, we also examined whether it could drive EMT in pluripotent stem cells. We showed that human ESCs, H1 transduced with virus encoding *SNAI1* exhibited rapid morphology change and down regulation of pluripotent genes such as *OCT4, SOX2* and *NANOG* (Fig. 4E). Also, the epithelial genes like *EPCAM* and *CDH1* were suppressed, while the mesenchymal genes were up-regulated (Fig. 4E), suggesting that *SNAI1* induced the differentiation by promote EMT in human ESCs. Taken all the data together, we demonstrate that *SNAI1* is one of the key downstream effector of TGFβ signaling that negatively regulate reprogramming and pluripotency.

## Discussion

In previous report, we had shown that HUCs could be directly converted into NPCs by the typical reprogramming factors combined with a cocktail of small molecules in medium. However, how these chemicals switched OSKM induced cell fate from iPSCs to NPCs remain unknown. In this report, we showed that A8301, a selective inhibitor for TGFβ signaling was sufficient to switch the cell fate to NPCs in factors (OSKM)-induced reprogramming of HUCs. Then through removing TGFβ, we further developed an improved approach in using simplified, totally defined medium (E7) for conversion of HUCs into NPCs. Moreover, we showed that TGFβ plays distinct roles at early and later stage of OSKM induced HUCs reprogramming. Early exposure of TGFβ suppressed reprogramming by activation of *SNAI1* and EMT. While at later stage, it’s required for establishing pluripotency through activation of *OCT4* and *NANOG*. These findings are consistent to previous reports in other cell types^18^,^22^,^23^. These data provide us a rationale for developing efficient approaches for generation both iPSCs and NPCs from HUCs. This is particularly important for non-vial approaches as many non-viral methods for human cell reprogramming displayed very low efficiency. Indeed, through manipulating TGFβ, we could generate both NPCs and iPSCs through one round of reprogramming from the same batch of HUCs, which is much timing and labor effective (supplementary Fig. S3), and would be valuable to generate patient-specific NPCs or iPSCs for disease modeling or drug screening.

TGFβ signaling has been known as an important morphogene for cell fate determination during embryonic development^24^,^25^ and also plays important role in reprogramming^19^,^21^. Mechanistically, TGFβ could induce EMT as a barrier for somatic cell reprogramming^18^,^19^. On the other hand, TGFβ is required for human ESCs or iPSCs to maintain self-renewal through directly target pluripotent genes, such as *NANOG*^26^,^27^,^28^,^29^. The origin cell type for reprogramming may undergo different molecular and cell changes induced by TGFβ and *SNAI1* activation, the exact underlying mechanism on how TGFβ coordinate with OSKM for fate decision during HUCs reprogramming is not clear at this time. We observed that the activation of endogenous *SOX2* did not rely on TGFβ signaling (Figs 2B and 3G), future studies will need to investigate how OSKM and TGFβ signaling differentially regulate these critical genes. Besides, during the preparation of this manuscript, two other reports published showing that OSKM induced somatic lineages such as cardiomyocytes or NPCs from mouse fibroblast were actually originated from a transient and unstable pluripotent state^30^,^31^. However, by using RT-PCR, we failed to detect the expression of endogenous *OCT4* during HUCs reprogramming in the absence of TGFβ signaling, indicating the different mechanisms in generating different lineages.

## Methods

All experiments were carried out in accordance with the guidelines of the Human Subject Research Ethics Committee at Guangzhou Institutes of Biomedicine and Health (GIBH), Chinese Academy of Sciences (CAS), and the Committee approved the experiments. Formal informed consent was obtained from all subjects.

### Cell culture and reprogramming

Human urine cells were isolated by the protocol reported in our previous publication^32^. The primary urine cells were cultured in urine cell medium consisting of a 1:1 mixture of DMEM/F12 culture medium supplemented with 10% of fetal bovine serum (FBS, PAA), 0.1 mM nonessential amino acids (NEAA), 1 mM L-glutamax, 0.1 mM β-mercaptoethanol and SingleQuot Kit CC-4127 REGM (Lonza). For reprogramming, urine cells were directly plated to Matrigel-coated 6 well plate (1−3 × 10^5^ cells per well) in urine cells culture medium. The vectors containing *OCT4, SOX2, KLF4* and *MYC* genes named psin-OS and psin-KM were transfected into urine cells by lentivirus. On day 2 after infection, the urine cells culture medium was changed into different reprogramming medium with different inhibitors. The inhibitors were 0.5 μM A8301(A), 1 μM PD032590(P), 3 μM CHIR99021(C), and 0.5 μM thiazovivin(T). The basal mediums were mTeSR1, E7 or E8 medium. The medium was changed every 2 days during the induction period. Colonies were counted in 18 days after infection, and picked up for identification or further culture in 21 days after infection. UiNPC colonies that possess polarity arrangement including rosette or neural-tube like structure were cultured in N2B27 medium of a 1:1 mixture of DMEM/F12 supplemented with 1% N2 (Invitrogen) and Neurobasal medium supplemented with 2% B27 (Invitrogen) supplemented with 20 ng/ml bFGF, 20 ng/ml EGF to form UiNPC spheres.

### Neuronal differentiation

UiNPCs were propagated in N2B27 medium with EGF and bFGF. For pan-neuronal differentiation, UiNPC spheres were then plated on Matrigel-coated coverslips and subsequently supplemented with N2B27 medium withdrawal of EGF and bFGF, and addition of neurotrophic factors, 1 μM dbcAMP and BDNF, GDNF, CNTF (both at 10 ng/ml, Peprotech) to aid in neuronal survival. All media were replenished at least once every 2d. UiNPCs differentiated for 2 weeks were examined for expression of neuronal markers and astrocyte marker.

### Quantitative RT-PCR (qRT-PCR)

Total RNA was extracted using TRIzol (Invitrogen). Quantitative real time RT-PCR (qPCR) was performed using a Thermal Cycler DiceTM Real Time System and SYBR Premix EX TaqTM (Takara). *ACTIN* was used for qPCR normalization, and all items were measured in triplicate. All primer sequences are listed in Table S1.

### Immunocytochemical analysis

Cells were fixed in 4% p-formaldehyde dissolved in 0.1 M phosphate buffer (PB) for 20 min. After several washes with 0.01 M phosphate-buffered saline (PBS), the cultures were incubated with the primary antibodies in PBS plus 1% BSA, 10% normal goat serum, and 0.3% Triton X-100 over night at 4 °C. The primary antibodies were listed in Table S2. Primary antibodies were visualized with species-specific secondary antibody conjugated to the fluorescent labels Alexa 568 or 488 (1: 400; Invitrogen). Cells were mounted in anti-fade medium containing 4′, 6-diamidino-2-phenylindole (Sigma) to counterstain nuclei. Cells were imaged on 710 NLO two photon confocal microscopy (Zeiss).

### Electrophysiological analysis

Whole-cell patch-clamp recording techniques were used to study the intrinsic properties of UiNPC-derived neurons in culture. Patch pipettes (resistance 3−5 MΩ) were filled with the following (in mM): 140 potassium methanesulfonate, 10 HEPES, 5 NaCl, 1 CaCl_2_, 0.2 EGTA, 3 ATP-Na_2_, 0.4 GTP-Na_2_, pH 7.3 (adjusted with KOH). The external solution contained (in mM): 120 NaCl, 1.2 KH_2_PO_4_, 1.9 KCl, 26 NaHCO_3_, 2.2 CaCl_2_, 1.4 MgSO_4_, 10 D-glucose, 7.5 HEPES (pH with NaOH to 7.3). The bath solution was equilibrated with 95% O_2_ and 5% CO_2_ before use. Resting potentials were maintained at about −60 mV. Whole-cell patch-clamp recordings were amplified and filtered using an Axopatch 200 B amplifier (Molecular Devices, Sunnyvale, CA). Signals were sampled at 10 kHz using a Digidata1440A analog-to-digital converter and acquired and stored on a computer hard drive using pClamp10 software. Data were analyzed using pClamp10 (Clampfit).

### Gene overexpression

To generate *SNAI1* overexpressing constructs, lentiviruses for gene expression were generated with psin-puro vector. To produce infectious lentiviral particles, HEK293T cells cultured on 10 cm dishes were transfected with target plasmids together with the packaging plasmids psPAX2 and pMD2.G. using calcium phosphate transfection method. The medium was replaced the following day, and 2 days later viral supernatants were collected, centrifuged at 300 g for 5 min at 4 °C and filter sterilized through a 0.22 μm filter to remove cell debris. Virus was concentrated about 200-fold by ultracentrifugation at 25,000 rpm for 120 minutes at 4 °C and used immediately. These supernatants were administered in HUCs, ESCs and iPSCs. After infection, cells were selected in puromycin for 3 days.

### RNA sequence analysis

After the digestion of cultured HUCs, HUCs or *SNAI1* overexpressed HUCs cultured in E7 medium for 6 days after OSKM transfection, the target cells were pelleted and lysed with 200 ul Trizol (Invitrogen). Total RNA was prepared with Direct-zol RNA MiniPrep kit (Zymo Research) following the manufacture’s protocol. RNA was then purified, fragmented, reverse transcribed, labeled and amplified to generate sequencing-ready cDNA library with TruSeq RNA Sample Prep Kit (Illumina). A size selection step was included to purify cDNA libraries to enrich for 250–300 bp fragments instead of AMPure XP beads purification. The DNA was recovered from each gel slice using QIA quick gel extraction kit (QIAGEN). The cDNA library concentration was determined with Qubit dsDNA HS Assay kit (Invitrogen). Additional sample concentrating step was included if the library concentration falls below required loading amount. The samples were run on MiSeq system with MiSeq Reagent Kits v2 (50 cycles) (Illumina). In data analysis, the correlation analysis, to avoid TPM divide by zero errors, log (0) errors, 1 is added to the TPM value and then log transform the expression value. In differential expression profiles analysis, The up-regulated genes in UC OSKM E7 or UC OSKM E7 *SNAI1* samples were those with fold change >2, the down-regulated genes in samples were those with fold change <1/2.

### Bisulfite sequencing PCR, BSP

Digest 100 ng genomic DNA with 0.5 μl BamHI (NEB) and 0.5 μl XhoI (NEB) in 37 °C 6 h; Boiling the tube in water for 10 min and place it immediately on ice for 5 min; Add 4 μl 2 M NaOH (Guangzhou chemical reagent factory) in the tube, 50 °C 15 min; Add 50 μl 2% LMP (low melting point agarose, Yesen) to the DNA mixture, Add 11 μl DNA/LMP mixture to 300 μl precooling Mineral oil(Beyotime). The mixture will form a small bead sink to the bottom; Place the EP tube on ice for 15 min. Add 500 μl 2.5 M sodium metabisulfite solution in the tube. Incubate the tube in 50 °C for 12 h, (pay attention to avoid light). Remove the supernatant in the EP tube and wash the beads 3 times with 1 ml PH8.0 TE to remove the remaining bisulfite, each time 10 min; Wash the beads 2 times with 500 μl 0.2 M NaOH, each time 15 min; Wash the beads 3 times with TE, each time 10 min; Wash the beads 2 times with ddH_2_O; use the beads as templates to amplify the target promoter region with the specific PCR primers (Table S1). After 2 rounds of PCR, the product will be cloned into PMD 18-T Vector (TaKaRa), and will be sequenced. The sequence data will be analysis and form the final result.

## Statistics

Data were compared using standard or repeated measures, using ANOVA where appropriate. Pair wise comparisons were performed using a two-tailed Student’s *t* test. For all data, differences were considered to be significant for *p* < *0.05*.

## Additional Information

**How to cite this article**: Wang, L. *et al*. TGFβ signaling regulates the choice between pluripotent and neural fates during reprogramming of human urine derived cells. *Sci. Rep*. **6**, 22484; doi: 10.1038/srep22484 (2016).

## Supplementary Material

Supplementary Information
