# Supplementary material for: TGFβ signaling regulates the choice between pluripotent and neural fates during reprogramming of human urine derived cells
Source: Sci Rep. 2016 Mar 3;6:22484. doi: 10.1038/srep22484 (PMC4776143; doi:10.1038/srep22484)
Supplement: Supplementary Information [file srep22484-s1.pdf]

Supplementary Information

TGFβ signaling regulates the choice between pluripotent and neural fates during reprogramming of human urine derived cells

Lihui Wang[1,2,+], Xirui Li[1,+], Wenhao Huang[1], Tiancheng Zhou[1], Haitao Wang[1], Aiping Lin[1], Andrew Paul Hutchins[1], Zhenghui Su[1], Qianyu Chen[1], Duanqing Pei[1], Guangjin Pan[1,*]

[1]Key Laboratory of Regenerative Biology, South China Institute for Stem Cell Biology and Regenerative Medicine, Guangzhou Institutes of Biomedicine and Health, Chinese Academy of Sciences, Guangzhou, China.

[2] Department of Pathology, Dalian Medical University, Dalian, China.

*Correspondence: Guangzhou Institutes of Biomedicine and Health, Chinese Academy of Sciences, Guangzhou, China, 510530. E-mail: pan_guangjin@gibh.ac.cn

[+] These authors contributed equally to this work.

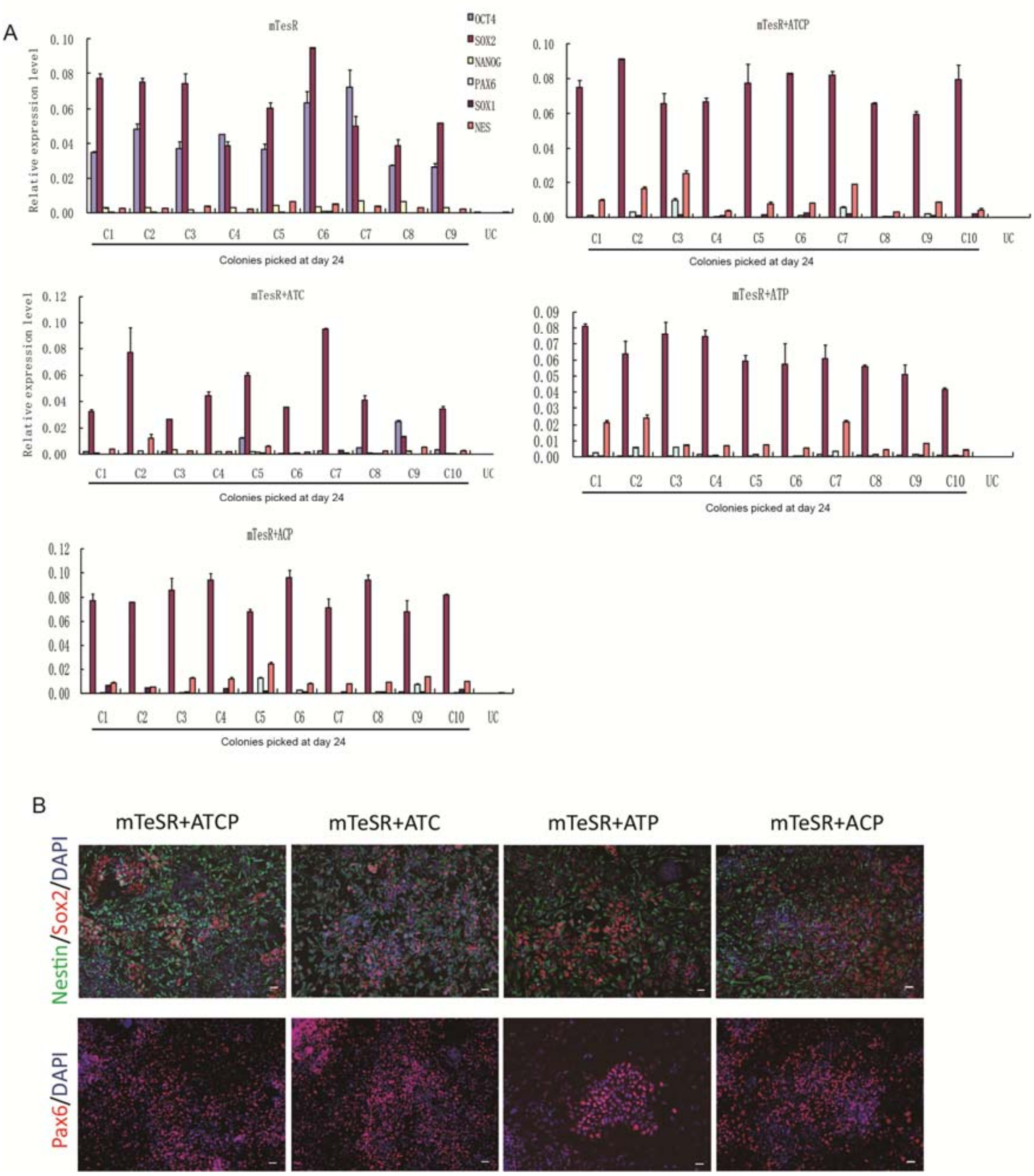

Fig. S1. The roles of 4i cocktail in cell fate switching during OSKM induced reprogramming of HUCs (A) A series of OSKM induced HUCs reprogramming in medium with one of each individual compound removed from the 4i cocktail. The colonies were randomly picked at day 24 and directly analyzed for endogenous pluripotent genes *OCT4*, *NANOG* and NSC genes *SOX2*, *SOX1*, *NES* and *PAX6* expressions by qRT-PCR. (B) Micrographs show immunostains of colonies for the indicated markers (D24). Scale bars: 50 μm. Error bars, s.d., n = 3 experiments.

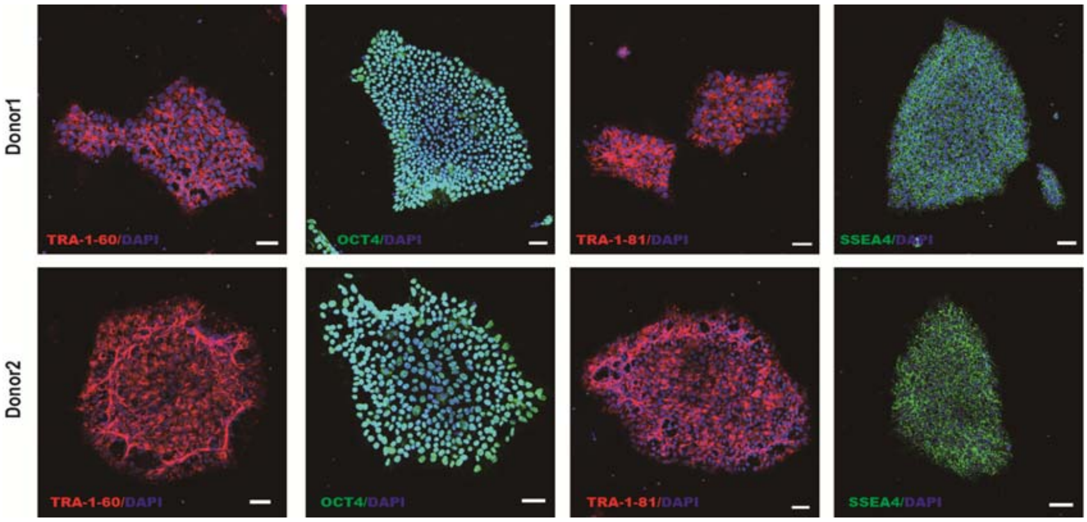

Fig. S2. Generation of iPSCs from HUCs by OSKM induced reprogramming with early inhibition and later introducing TGFβ Micrographs show immunostains of colonies cultured in the medium with introducing TGFβ at D6 of reprogramming for the pluripotent markers. Scale bars: 50 μm.

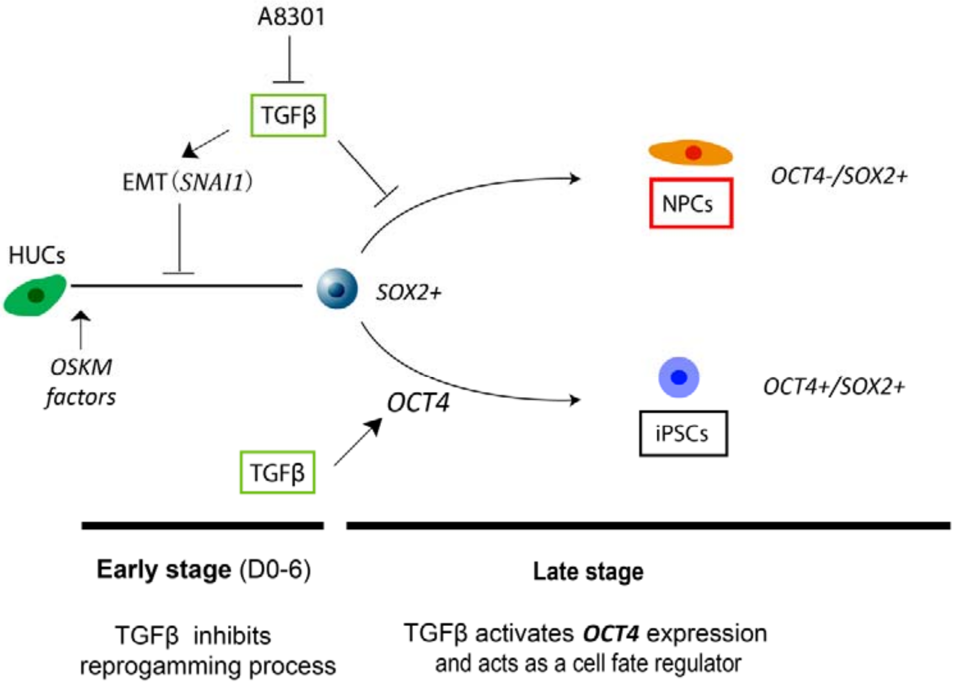

Fig. S3. Schematic picture illustrating the role of TGFβ in OSKM induced reprogramming of HUCs.

Table S1. Primer sets for qPCR and PCR

For qPCR		
Gene	*Sequences (5’ →3’)*	
ACTIN	*Forward*	*CCCAGAGCAAGAGAGG*
	Reverse	*GTCCAGACGCAGGATG*
SOX17	*Forward*	*GTGGACCGCACGGAATTTG*
	Reverse	*GGAGATTCACACCGGAGTCA*
T	*Forward*	*TATGAGCCTCGAATCCACATAGT*
	Reverse	*CCTCGTTCTGATAAGCAGTCAC*
SOX1	*Forward*	*AATTTTATTTTCGGCGTTGC*
	Reverse	*TGGGCTCTGTCTCTTAAATTTGT*
NES	*Forward*	*CTGGAGCAGGAGAAACAGG*
	Reverse	*TGGGAGCAAAGATCCAAGAC*
PAX6	*Forward*	*ATGTGTGAGTAAAATTCTGGGCA*
	Reverse	*GCTTACAACTTCTGGAGTCGCTA*
OCT4	*Forward*	*CCTCACTTCACTGCACTGTA*
	Reverse	*CAGGTTTTCTTTCCCTAGCT*
NANOG	*Forward*	*TGAACCTCAGCTACAAACAG*
	Reverse	*TGGTGGTAGGAAGAGTAAAG*
SOX2	*Forward*	*CCCAGCAGACTTCACATGT*
	Reverse	*CCTCCCATTTCCCTCGTTTT*
SNAI1	*Forward*	*AATCGGAAGCCTAACTACAGCG*
	Reverse	*GTCCCAGATGAGCATTGGCA*
SNAI2	*Forward*	*CATGCCTGTCATACCACAAC*
	Reverse	*GGTGTCAGATGGAGGAGGG*
CDH1	*Forward*	*TGCCCAGAAAATGAAAAAGG*
	Reverse	*GTGTATGTGGCAATGCGTTC*
CDH2	*Forward*	*TGGGAATCCGACGAATGG*
	Reverse	*TGCAGATCGGACCGGATACT*
ZEB1	*Forward*	*AGCAGTGAAAGAGAAGGGAATGC*
	Reverse	*GGTCCTCTTCAGGTGCCTCAG*
TWIST1	*Forward*	*GGAGTCCGCAGTCTTACGAG*
	Reverse	*TCTGGAGGACCTGGTAGAGG*
EPCAM	*Forward*	*CTCCTTCTGAAGTGCAGTCCG*
	Reverse	*CTGCTCTGAGCGAGTGAGAACC*
FN1	*Forward*	*GAGCTATTCCCTGCACCTGATG*
	Reverse	*CGTGCAAGGCAACCACACT*
VIM	*Forward*	*GGAAGAGAACTTTGCCGTTGAA*
	Reverse	*GTGACGAGCCATTTCCTCCTT*
For PCR		
SNAI1	*Forward*	*CGGAATTCATGCCGCGCTCTTTCCTCG*
	Reverse	*CGGGATCCTCAGCGGGGACATCCTGAGC*
OCT4 promoter meth-1	*Forward*	*AGGTGTGGGAGTGATTTTAGATAGT*
	Reverse	*AAACCTTAAAAACTTAACCAAATC*
OCT4 promoter meth-2	*Forward*	*GAGGTTGGAGTAGAAGGATTGTTTTGG*
	Reverse	*CCCCCCTAACCCATCACCTCCACCACC*

Table S2. Primary antibody list

Antibodies	Source	Cat#	Dilution
Rabbit anti-Nestin	Millipore	AB5922	1:1000
Mouse anti-Pax6	Developmental Studies Hybridoma Bank	Pax6	1:1,000
Mouse anti-Sox2	R&D Systems	MAB2018	1:100
Rabbit anti-Sox1	Millipore	AB15766	1:1000
Mouse anti-Oct3/4	Santa Cruz Biotechnology	sc-5279	1:50
Rabbit anti-Glutamine	Sigma	G6642	1:5000
Rabbit anti-GABA	Sigma	A2052	1:2000
Mouse anti-Map2	Millipore	MAB3418	1:1000
Rabbit anti-Tuj1	Convance	PRB-435P	1:1,000
Mouse anti-Tuj1	Sigma	T8578	1:5000
Mouse anti-TH	Millipore	MAB318	1:500
Rabbit anti-GFAP	Sigma	SAB4501162	1:1000
Mouse anti-TRA-1–60	Millipore	MAB4360	1:100
Mouse anti- TRA-1–81	Millipore	MAB4381	1:50
Mouse anti-Nanog	R&D Systems	AF1997	1:20
Mouse anti-SSEA-4	Abcam	ab16287	1:50
